# How can General Practice be incorporated longitudinally in medical studies? Students’ views on the development of a new rural health program

**DOI:** 10.3205/zma001188

**Published:** 2018-08-15

**Authors:** Linda Barthen, Gisela Ravens-Taeuber, Michael A. Paulitsch, Ferdinand M. Gerlach, Monika Sennekamp

**Affiliations:** 1 Goethe University Frankfurt, Institute of General Practice, Frankfurt/Main, Germany

**Keywords:** general practice, medical students, curriculum, rural health program, shortage of family doctors

## Abstract

**Aim: **Participation of medical students in the conceptual development of targeted and attractive teaching content for rural areas.

**Method: **A questionnaire was developed to gather information on students' views of their current medical studies, career interests, and what requirements should be met by an optional rural health program in general practice. By means of an online survey in summer 2015, all medical students from the fourth preclinical semester onwards (n=2,150) at Goethe University Frankfurt were surveyed on one occasion. Statistical analysis was mainly descriptive. Personal attitudes towards a career as a family practitioner were examined for statistical significance. Further information was gathered on whether a measurable correlation exists between personal background and desired work location.

**Results: **Of the 2,150 students that were contacted, 617 participated in the survey (response rate=28.7%). The results covered a wide range of ideas and recommendations and were representative both of medical students with a positive attitude toward general practice, as well as those that were rather critical of teaching in general practice. The students expected the planned health program to be of strong practical relevance and to acquaint them with the administrative and economic aspects of running a practice.

**Conclusions:** By including the target group in the development process, it was possible to tailor the health program to meet the needs of future participants more precisely. Student participation can also be expected to result in greater acceptance of the program. The results on teaching content may also provide other medical faculties with orientation when developing comparable programs.

## Zusammenfassung

**Zielsetzung:** Beteiligung von Medizinstudierenden im Rahmen der konzeptionellen Entwicklung eines zielgruppenspezifischen und attraktiven allgemeinmedizinischen Lehrangebots im ländlichen Raum.

**Methodik: **Es wurde ein Fragebogen entwickelt, der die Bewertung der Studierenden hinsichtlich des aktuellen Ablaufs ihres Studiums, den späteren Berufswunsch sowie die Anforderungen an ein zu entwickelndes allgemeinmedizinisches Schwerpunktprogramm im ländlichen Raum erfasst. Mittels einer Online-Befragung wurden im Sommer 2015 alle Medizinstudierende ab dem vierten vorklinischen Semester (n=2.150) der Goethe-Universität Frankfurt einmalig befragt. Die statistische Auswertung erfolgte primär deskriptiv. Die persönliche Einstellung hinsichtlich der Bereitschaft, als Hausarzt tätig zu werden, wurde auf statistische Signifikanz überprüft. Zudem wurde erhoben, ob ein messbarer Zusammenhang zwischen der eigenen Herkunft und dem späteren Wunscharbeitsort besteht.

**Ergebnisse: **Von insgesamt 2.150 kontaktierten Studierenden nahmen 617 an der Befragung teil (Rücklaufquote=28,7%). Die Ergebnisse repräsentieren eine große Bandbreite an Ideen und Anregungen, die sowohl die Meinung von Befürwortern als auch eher kritisch gegenüber der Lehre in der Allgemeinmedizin eingestellten Medizinstudierenden widerspiegeln. Von dem geplanten Schwerpunktprogramm erwarten die Studierenden einen starken Praxisbezug ebenso wie das Kennenlernen administrativer sowie wirtschaftlicher Hintergründe zum Führen einer Praxis.

**Schlussfolgerungen:** Durch die Einbeziehung der Zielgruppe am Entwicklungsprozess bestand die Möglichkeit, das zu entwickelnde Schwerpunktprogramm auf die späteren Teilnehmer passgenauer zuzuschneiden. Zudem ist zu erwarten, dass die Beteiligung der Studierenden zu einer höheren Akzeptanz des Programms führt. Die gewonnenen Ergebnisse zur Gestaltung eines Lehrangebots können als Orientierung für die mögliche Entwicklung ähnlicher Schwerpunktprogramme an anderen medizinischen Fakultäten dienen.

## Introduction

Against the background of an increasing shortage of GPs, it is essential to attract a new generation of family doctors, particularly to the rural areas of Germany. The use of designated health programs during medical studies to promote general practice can have a positive effect on readiness to work as a family doctor (in rural areas) after completion of studies [1]. In foreign countries, and particularly in the U.S., Canada and Australia, numerous health programs already exist that are aimed at promoting general practice during medical studies [2], [3]. The number of such programs is also increasing in Germany [4], [5]. There are signs that the acquisition of skills during special general practice programs has a positive influence on the likelihood that a student will choose to specialize in general practice [6], and thus promotes general practice as a preferred career option [7].

The Faculty of Medicine in Frankfurt am Main has provided the "Fulda rural outing" since 2012. The outing is a voluntary support program that enables participating students to complete their general practice internship in selected rural practices in the district of Fulda in the Federal state of Hesse. It could be demonstrated that participation in the program significantly increased interest in later becoming a family doctor [8]. Faculty members and students both recommend longitudinally incorporating general practice in medical studies [9], [10]. For this reason, an expanded program is to be developed in cooperation with the Office of the Dean of Studies and provided in addition to the “Fulda rural outing”. The new program is to run for several semesters and involve further districts.

Although studies have shown that the participation of the target group is essential if a change to the curriculum is to be accepted and successful, students are seldom involved when curricular changes and innovations of this kind are planned [11]. As experts in matters affecting them personally, students know where the deficits and potential improvements in the current curriculum are, and are very interested in being involved in the change process [12]. 

The aim of the survey presented here was to include students in the conceptual development of a new “rural health program” (also to be referred to as “priority program”). The focus lay on the question what the students wanted and expected of a priority program aimed at promoting general practice in rural areas. Furthermore, information was collected on the students' desired careers and their evaluation of general practice as part of their medical studies.

## Methods

### Data collection 

An invitation to participate in a web-based survey with a cross-sectional design was sent to all students in their fourth preclinical semester or above and studying at Goethe University Frankfurt. They were contacted via the e-mail distribution lists of the Dean’s Office and the Institute of General Practice. The choice of student population was based on the assumption that from the fourth preclinical semester onwards students had sufficient knowledge of the way their course was structured and what awaited them in the future. Furthermore, e-mail distribution lists existed for these students, so direct contact was possible. The e-mail included a short information letter and the link to the online-questionnaire. At the end of May, the entire population of 2,150 students was contacted for the first time. A reminder was sent mid-June 2015. 

#### Survey instrument

The specially designed questionnaire (see attachment 1 ) was based on the results of a nationwide survey of medical students that took place in the year 2015 [13] and was further developed and agreed upon by an interdisciplinary team consisting of a physician in specialist training, an experienced GP and specialists in educational science, psychology and the health sciences.

The questionnaire was tested on three students and subsequently adapted. The final version of the questionnaire included 19 items broken down into four thematic sections –* socio-demographic information, career choice, medical studies, and comments on the rural health program to be developed*. They were also asked to gauge their inclination to set up as a GP in private practice on a six-point scale (1=“True”; 6=“False”). In addition to mostly closed questions, the questionnaire included five open questions. 

In the final thematic section, the participants were first presented with an initial idea of what the future priority program might look like, based on the literature and other established programs (components: training in a practice, accompanying seminar, and mentoring program [z.B. [14], [15], [16], [17]. Participants were subsequently required to comment on the outlined idea and then to say whether they would take part in such a program.

The Survey Monkey tool was used to ensure low-threshold access. In this way, it was hoped that students that had shown little or no interest in general practice to date, or were critical of the discipline, could also be encouraged to participate. 

The ideas for the rural health program that stemmed from the survey were agreed upon in a subsequent discussion session with individual faculty students. 

#### Data analysis

Data were mostly descriptively analyzed using the IBM SPSS statistics software. Absolute and relative frequencies were calculated for categorical variables. Possible differences in average values for readiness to work as a GP were tested for statistical significance using a paired Wilcoxon test. The effect size of any differences were quantified by calculating Cohen's d: 0.2–0.4=small effect; 0.5–0.8=moderate effect and >0.8=large effect [18]. In addition, any correlation between background and desired place of work was tested using Spearman’s correlation coefficient (α≤5%).

Socio-demographic information was presented by reducing the six-levels of the rating scale on personal background to a dichotomous *rural community* or *town* answer [19]. 

Free-text answers were analyzed using quantitative content analysis, with relevant attributes of text passages being identified and operationalized via allocation to a developed system of categories. Frequencies of allocation to individual categories were regarded as indicators of text attributes [20]. Participants in the survey generally answered in whole sentences, or by naming key points. Single points were regarded as single units (e.g. question 13 a *“Receive own patients for a preliminary investigation”*), longer presentations of key points or several sentences were divided into several units, depending on content (e.g. question 13 a *“Practice should make at least one investigation room available to students, and it would also be good to be able to perform instrument-based diagnostics”*. The individual units were then allocated to the categories (e.g. *“Receive own patients for a preliminary investigation”*=Do practical work; *“Practice should make at least one investigation room available to students”*=Own treatment room; *“… instrument-based diagnostics…”* Get to know and use instrumental diagnostic procedures) that were previously derived from the text material (inductive approach). Two persons were used to analyze and encode the open texts. Only those categories that were named at least 10 times were presented in the results (see table 1 (Tab. 1)).

## Results

After cleansing the data, 617 questionnaires were suitable for analysis (response rate=28.7%). 423 participants were female (68.6%). The median year of birth was 1990 (range: 1968-1995), and on average the participants were in their 8^th^ semester (range: 2^nd ^– 16^th^ semester; one person said they were in their 2^nd^ semester). Most participants grew up in a town (>5,000 inhabitants). Around one fifth (21.0%) came from a rural community (<5,000 inhabitants). Furthermore, nearly one fifth (18.5%) said they had completed vocational training in another profession. Only 4.5% had children. 

### Current study situation and career choice

When asked at what stage in their studies medical students should gain their first practical experience in a family practice, almost one quarter (23.5%) said it should be in the preclinical phase. A clear majority of nearly two-thirds (65.8%) regarded the clinical stage of their studies as the right time. Only 5.4% were in favor of such practical training before beginning their studies. Differences existed between students in preclinical and clinical stages of their studies, with 41.7% of those in the preclinical phase regarding the preclinical phase as suitable, while only 17.6% of those in the clinical phase did. 

The questionnaire did not collect data on students' experience of general practice, but responses could be expected to depend on the year of studies a student was in. 

Students that considered a career in general practice to be possible were asked whether they thought their education to date had prepared them sufficiently. The further a student had progressed in their studies, the more likely the answer was to be yes. Nonetheless, 19.0% of respondents from the 11^th^ semester onwards (N=158) said they did not feel sufficiently prepared, and 55.1% said they were only partially prepared (74.1% overall). The respondents said that in order to feel sufficiently prepared (N=97; open question), they would need *more practical experience* (53.6%), to be *acquainted with the administrative and economic aspects of running a practice* (15.5%), and *more interdisciplinary knowledge* (11.4%).

A basic interest in working in family medicine was also measured using a 6-point rating scale (1=“True”; 6=“Untrue”). The participants were asked whether* “at the beginning of their studies, they could imagine working as a family doctor in the future”* (average 3.81) and whether they *“could now imagine working as a family doctor in the future”* (average 3.36). A comparison of the results using the Wilcoxon test showed that interest increased statistically significantly during the course of studies (p<0.001; N=617). At 0.278, Cohen’s d showed a small effect size [18]. Furthermore, 43.5% of students (n=268) said they could *not really* or *not at all* imagine later working as a GP. Nevertheless, 46.7% of these (N=125) made specific proposals and had definite ideas on how to structure practical training. These results can be seen in Table 1.

**Figure 1:**
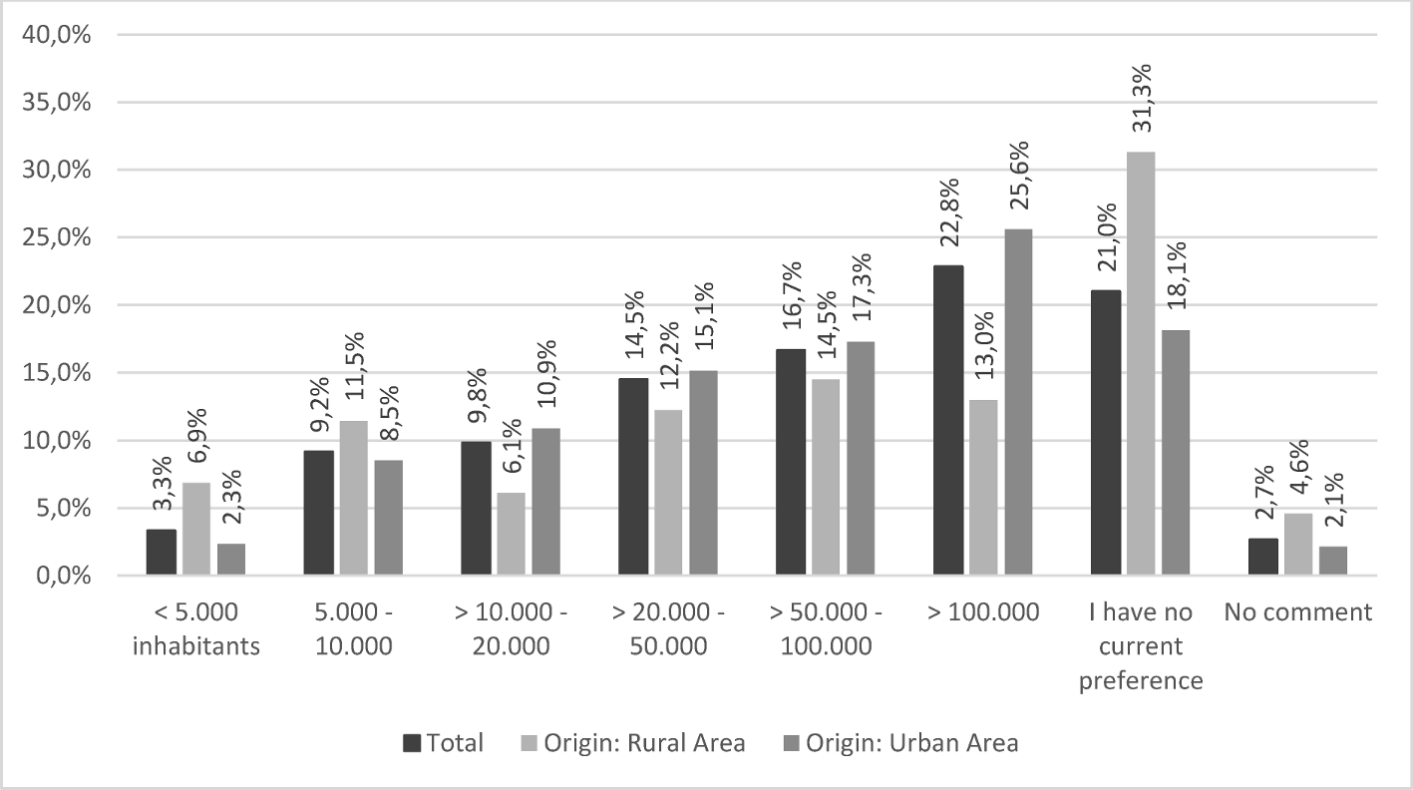
Preferred number of inhabitants living in future place of work according to background, N=617.

Figure 1 (Fig. 1) shows the preferred number of inhabitants living in the future place of work. A statistically significant correlation exists between the variables of own background and desired place of work with a moderate effect size (r=0.366; p<0.001; N=458) [20]. Almost one third of participants from rural communities said they had no preferences with regard to future place of work. 

#### Information on the planned rural health program

To structure the priority program, the students were first provided with a preliminary concept (part I: training in a practice; part II: accompanying seminar; part III: mentoring program). Around one half of participants took the opportunity to formulate their own requirements of a rural health program in general practice and express their own ideas in free text. 

62.9% of participants mentioned *active practice involvement *as the most important requirement of training in a family practice (part 1). This predominantly involved treating their own patients independently, from taking a medical history through diagnostic investigations to making a therapy recommendation (initially) under supervision. In addition, more than a third of participants (35%) wanted *regular and detailed feedback from the teaching physician*. With regard to accompanying seminars in small groups at the university (part II), the students mainly mentioned specific content for inclusion in the seminars. Practical exercises (42.6%) and discussion of experience and cases after the practice phase (29.0%) were considered particularly important. The participants expected of mentoring (part III) that a family practitioner should provide them with theoretical and practical tips and feedback (23.6%) and show a readiness to answer all types of question (25.1%). The students hoped the mentoring would enable them to develop practical skills, for example by means of practical training (27.0%). Complete results can be found in table 1 (Tab. 1).

Students’ responses on the desired length of the health program varied (answers were predefined). More than a quarter (27.7%) were in favor of one semester, with a two further quarters either wanting it to last several semesters (23.0%), or not specifying a length as long as the *“course is useful to me”* (23.5%). Fewer than 5% wanted a priority program that continued throughout the entire length of medical studies. 

Finally, the participants were asked whether they would participate in the outlined course (yes=45.4%; no=17.5%; don’t know=30.6%; no response=6.5%). Those that were against participation attributed their decision to *“no interest in general practice”* (61.5%),* “no interest in working in a rural area”* (45.0%), *“no time during studi*es” (41.3%), *“no interest in setting up in private practice”* (21.1%), and *“no interest in further practical training”* (21.1%).

## Discussion

617 medical students from the fourth preclinical semester onwards used the online questionnaire to participate in the development of a general practice priority program for rural areas. 

The need for more practical training in medical studies is reflected in the results of the present study and the requirements that students thought the planned rural health program should satisfy. The potential participants saw a need for greater practical orientation in all three core areas (training in a practice, accompanying seminar, and mentoring program). An interest in treating patients, further developing personal skills and being actively involved in day-to-day practice life was mentioned in the open questions. Furthermore, it was seen as important to be familiarized with practice management and practice organization. This is therefore a field that should be considered in the priority program. 

Regardless of specialization, more than half the participants (54.0%) would prefer to work in a mid-sized or large town (>20.000 inhabitants). It is unclear whether this rules out “living in a town and working in the country” as a possible model for the future. It has often been noted that students with a rural background often show a greater readiness to return to the country [1], [21]. The fact that almost a third (31.3%) of students with a rural background are undecided as to where they would like to work, indicates that this target group should be encouraged to participate in the priority program in order to awaken their interest in a career in a rural region early on. 

Almost three-quarters of all participants (74.1%) from the 11th semester onwards did not feel they had been prepared or adequately prepared for a future career in general practice. The reasons for this are often a lack of practical experience, but also insufficient knowledge of the administrative and economic know-how required to run a practice. The results present no reason for alarm, as specialist training in general practice is supposed to fulfil these needs. However, the aim of medical studies is *“… to teach basic knowledge, skills and proficiency in all the disciplines that are necessary to provide comprehensive healthcare to the population”* [https://www.gesetze-im-internet.de/_appro_2002/BJNR240500002.html]. Nonetheless, a survey of graduates was able to show that the lack of practical orientation and insufficient teaching of practical, medical skills are seen as weaknesses of medical studies [22]. Policymakers have also recognized these problems and are now actively trying to do something about them as part of the Masterplan Medical Studies 2020. The intention of the Masterplan is not only to raise the share of general practice in medical studies, but also to strengthen training in a family practice setting [23]. 

The investigation was also able to show that students' interest in possibly working in general practice increased during the course of their studies. A 2015 study carried out by Jacob, Kopp und Schultz came to a similar conclusion [13]. It should further be noted that although the rural health program could only be described in broad outlines at the time of the survey, and it is extremely specialized (general practice in rural areas), 45.4% of participants were able to imagine taking part in it. Although it cannot be assumed that actual interest will be correspondingly high, it is obvious that there is a need for such a program and that it makes sense to develop one.

The results presented here have some limitations. The questionnaire was developed specifically for the study and has not been validated. It should also be taken into consideration that cross-sectional surveys are not able to assess longitudinal changes that occur in students during the course of their studies, so divergences between cohorts in successive years may be due to factors that were not revealed here. As far as transferring the approach and the developed concept to other German study locations is concerned, it should be borne in mind that conditions differ between universities. The present results are therefore not representative for Germany as a whole and implementation elsewhere should be adapted to take account of local circumstances. On the other hand, one strength of the study is the participation of students in the development of the curriculum. It could be shown that students that were critical of general practice, or that had no interest in a career in family medicine, also participated in the survey. A broad range of views could thus be captured, making our approach preferable to the use of focus groups, in which those with no interest in general practice would not have participated. Nevertheless, in order to benefit from student feedback, we presented and agreed upon the developed concept in a discussion group with individual faculty students. 

## Conclusions

Overall, the participation of students helped to give concrete form to the content, organization and thematic design of the country doctor program. Initial ideas could be further developed, new approaches generated and observations that had not previously been considered taken into account. For example, the decision not to provide the priority program before the clinical stage of medical studies was made on the basis of the results. Furthermore, issues concerning running a practice and practice management will be included in the course of seminars. At heart, the program will and must demonstrate continual practical relevance, which, among other things, will be manifested in practical exercises during the seminars. 

It is also to be expected that the inclusion of the target group will raise acceptance of the program. However, it may make sense in the future to consider finding ways to intensify and improve feedback from the students so their opinions continue to be reflected in the results. Overall, the methodological approach of involving students in the development of the curriculum can be recommended. The results can provide orientation to those involved in the development of rural health programs in other medical faculties.

## Acknowledgement

We would especially like to thank Inga Beig for her great help in entering the data. 

## Competing interests

The authors declare that they have no competing interests. 

## Supplementary Material

Questionnaire: Development of an optional rural health program

## Figures and Tables

**Table 1:**
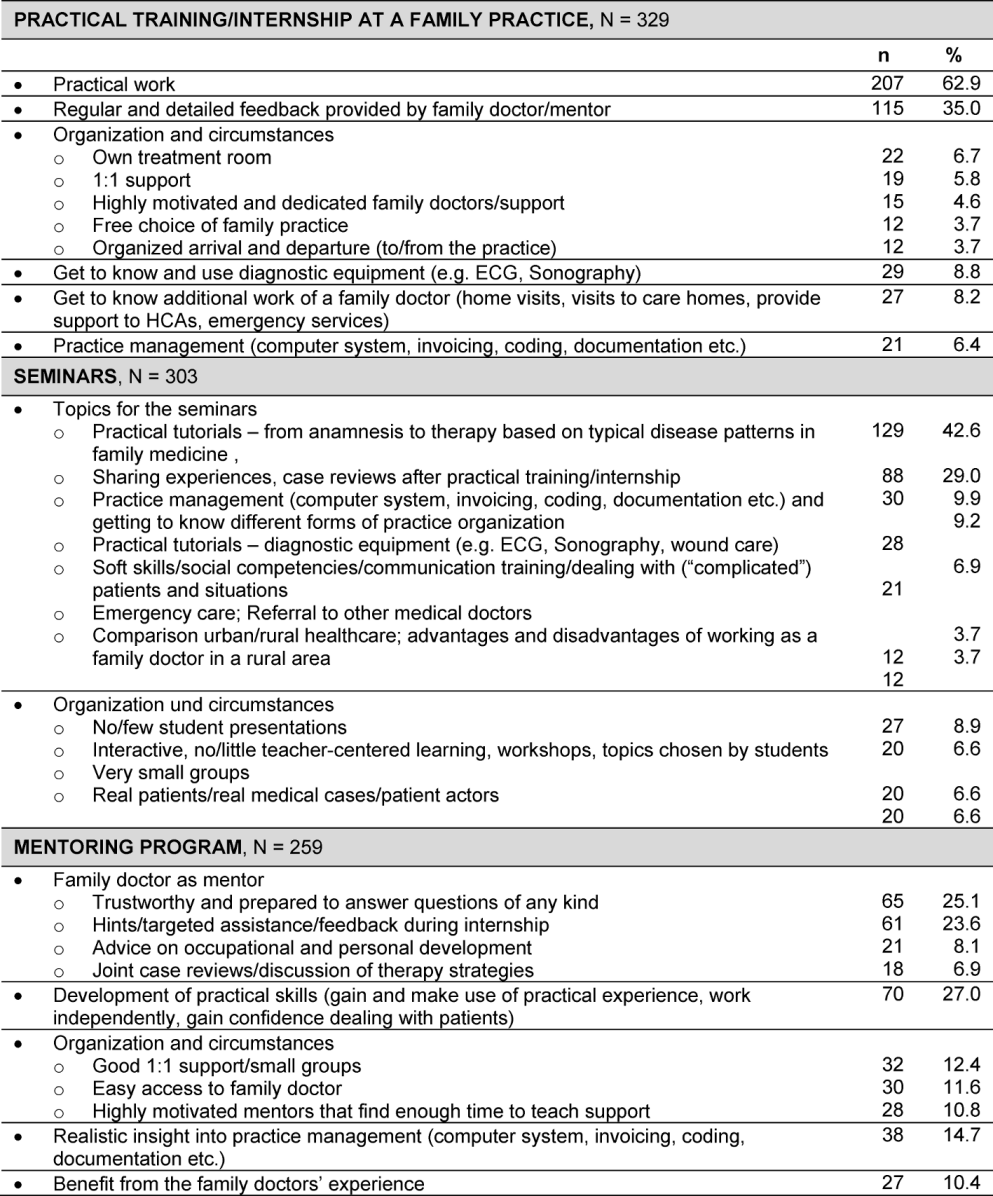
Information on the three elements of the rural health program. (Evaluation of open questions; Illustration of categories mentioned at least 10 times).
