# Supplementary material for: How can General Practice be incorporated longitudinally in medical studies? Students’ views on the development of a new rural health program
Source: GMS J Med Educ. 2018 Aug 15;35(3):Doc42. doi: 10.3205/zma001188 (PMC6120159; doi:10.3205/zma001188)
Supplement: Questionnaire: Development of an optional rural health program [file JME-35-42-s-001.pdf]

Questionnaire

Development of an optional rural health program

General information

1) Year of birth ☐☐☐☐

2) Which semester are you in? ☐

3) Sex
- ☐ Female
- ☐ Male

4) How many people lived in the place you mainly grew up?
- ☐ < 5.000
- ☐ > 5.000 – 10.0000
- ☐ > 10.000 – 20.000
- ☐ > 20.000 – 50.000
- ☐ > 50.000 – 100.000
- ☐ > 100.000

5) Have you completed any other job training?
- ☐ Yes (Please specify): ____________________
- ☐ No

6) Do you have any children?
- ☐ Yes
- ☐ No

Career aspirations and academic studies

In this section we want to get to know about your career aspirations und your assessment of your current studies.

To what extent do the following statements apply to you?

7) <u>“At the beginning of my medical studies I could imagine working as a family doctor in the future.”</u>

True ☐ ☐ ☐ ☐ ☐ ☐ False

8) “<u>Now</u> I can imagine working as a family doctor in the future.”

True ☐ ☐ ☐ ☐ ☐ ☐ False

9) If you could choose your future place of work, how many people would live there?
- ☐ > 100.000
- ☐ > 50.000 – 100.000
- ☐ > 20.000 – 50.000
- ☐ > 10.000 – 20.000
- ☐ > 5.000 – 10.0000
- ☐ < 5.000
- ☐ I have no current preferences
- ☐ I don’t know

10) In your personal opinion: at what stage of medical studies should medical students first gain experience of working in a family practice?

- ☐ Before medical studies begin
- ☐ 1st semester (preclinic)
- ☐ 2sd semester (preclinic)
- ☐ 3rd semester (preclinic)
- ☐ 4th semester (preclinic)
- ☐ 5th semester (clinical)
- ☐ 6th semester (clinical)
- ☐ 7th semester (clinical)
- ☐ 8th semester (clinical)
- ☐ 9th semester (clinical)
- ☐ 10th semester (clinical)
- ☐ 11th semester (clinical)
- ☐ 12th semester (clinical)
- ☐ I don't know
- ☐ Another time: ______________________

11) Assuming you can imagine pursuing a career in family medicine in a rural area: Do you feel your medical studies have so far prepared you adequately?

- ☐ Yes
- ☐ No
- ☐ Partly
- ☐ It is too early to tell
- ☐ I don't know

12) <u>If you answered "No" or "Partly":</u> what would you require to feel adequately prepared to work as a family doctor in a rural area?

____

____

____

____

____

Information on the rural health program

PLEASE READ THE FOLLOWING INFORMATION CAREFULLY:

The optional program that is to be developed may contain the following:

1. **Practical training:** Working/Internship in a family practice in a rural area.
2. **Seminars in small groups:** e.g. before and after practical training, discussions with experts, presentations.
3. **Mentoring program:** longitudinal support from family doctors.

The goal of the program is to convey a realistic impression of the work of a family doctor in a rural area.

To minimize or avoid the need to find additional time for the program, we aim to make it a clinical elective course that will be included in your regular medical training.

It is also conceivable that other mandatory courses will be included in the program, e.g. preclinical elective, clinical traineeship, the first course in family medicine, the internship in family medicine and/or four months during the final "practical year".

13) Assuming you would participate in the rural health program: Do you have any ideas/suggestions/wishes …

a) … how should the PRACTICAL TRAINING (working/internship in a family practice in a rural area) be designed to ensure it is a useful addition to your current medical studies (content, organization etc.)?

____

____

____

____

____

b) … how should the SMALL GROUP SEMINARS be designed to ensure they are a useful addition to your current medical studies (topics, content, organization etc.)?

____

____

____

____

____

14) Would you be interested in a mentoring program (support provided by experienced family doctors)?

- ☐ Yes
- ☐ No
- ☐ I don't know

15) What would you expect of a mentoring program?

____

____

____

____

____

16) How long should the program, consisting of practical training, small group seminars and a mentoring program, last?

- ☐ One semester
- ☐ Several semesters
- ☐ Throughout the whole of medical studies
- ☐ Doesn't matter as long as it is useful to me
- ☐ I don't know
- ☐ Another period: ___________________________________

17) Would you participate in a rural health program, consisting of practical training, small group seminars and a mentoring program?

- ☐ Yes
- ☐ No
- ☐ I don't know

18) <u>If you answered "No", why not?</u>
Multiple answers possible.

- ☐ The content of the program is not clear enough. I can't imagine what is involved.
- ☐ I am not interested in family medicine.
- ☐ I am not interested in working in my own practice.
- ☐ I am not interested in working in a rural area.
- ☐ I am not interested in additional internships.
- ☐ Medical studies already provide all necessary information.
- ☐ No available time during my current medical studies.
- ☐ Others: ________________________________

19) Do you have any further suggestions/ideas:

____

____

____

____

____

Thank you for participating.
